# Polarity of the First Episode and Time to Diagnosis of Bipolar I Disorder

**DOI:** 10.4306/pi.2009.6.2.96

**Published:** 2009-06-30

**Authors:** Boseok Cha, Jeong Hyun Kim, Tae Hyon Ha, Jae Seung Chang, Kyooseob Ha

**Affiliations:** 1 Mood Disorders Clinic and Clinical Affective Neuroscience Laboratory, Department of Psychiatry, Seoul National University Bundang Hospital, Seongnam, Korea.; 2 Department of Psychiatry and Behavioral Science, Seoul National University College of Medicine, Seoul, Korea.

**Keywords:** Bipolar disorder, Polarity, First episode, Diagnosis, Suicide

## Abstract

**Objective:**

The current study explored the relationship between the polarity of the first episode and the timing of eventual diagnosis of bipolar I disorder, and associated clinical implications.

**Methods:**

Twelve years of clinical data from the medical records of 258 inpatients meeting DSM-III-R or DSM-IV criteria for bipolar I disorder were analyzed. Subjects were divided into two groups according to the polarity of the first episode: those with depressive polarity (FE-D), and those with manic polarity (FE-M). Comparisons were made between the two groups on variables associated with the timing of diagnosis and related outcomes.

**Results:**

In population with bipolar I disorder, a significant longer time lapse from the first major mood episode to the confirmed diagnosis was associated with the FE-D group compared to the FE-M group [5.6 (±6.1) vs. 2.5 (±5.5) years, p<0.001]. FE-D subjects tended to have prior diagnoses of schizophrenia and major depressive disorder while FE-M subjects tended to have prior diagnoses of bipolar disorder and schizophrenia. A significantly higher rate of suicide attempts was associated with the FE-D group compared to the FE-M group (12.7 vs. 1.7%, p<0.001).

**Conclusion:**

The results of this study indicate that first-episode depressive polarity is likely to be followed by a considerable delay until an eventual confirmed diagnosis of bipolar I disorder. Given that first-episode depressive patients are particularly vulnerable to unfavorable clinical outcomes such as suicide attempts, a more systematic approach is needed to differentiate bipolar disorder among depressed patients in their early stages.

## Introduction

A clinical diagnosis of bipolar disorder usually entails a relatively long interval between the initial presentation and the accurate diagnosis.^1^-^3^ Previous studies have reported that patients may wait for as long as 5-10 years^4^-^7^ from the onset of illness before the diagnosis is confirmed. Delays in the correct diagnosis and proper treatment of bipolar disorder may result in social, occupational, and economic burdens,^8^,^9^ as well as in an increase in completed suicides.^10^ Several factors underlie the lapse of time before diagnosis, including the relatively early onset of mood symptoms, the relative mildness of early symptoms of mood disorder,^11^ misdiagnosis as unipolar depression,^12^ and predominantly depressive presentation.^13^

When the first episode begins with depression, a rapid cycling course is more common;^14^ depressive episodes tend to dominate the subsequent course of the illness^15^,^16^ and suicide attempts are more common.^17^ Kassem et al.^18^ demonstrated that first-episode polarity tends to run in families, and is a useful way to identify patients with homogeneous subtypes of bipolar disorder. Nonetheless, few studies investigate the effects of first-episode polarity on the clinical course of bipolar disorder, which can include delays in diagnoses and suicide.

Since the diagnosis based on DSM-IV criteria mandates the evidence of manic/hypomanic episodes, it is not surprising that detecting bipolarity is often delayed in patients with depressive onset compared to those with manic onset. The present study addresses an open question in the literature regarding the quantification of the time to the correct diagnosis between patients with depressive onset and those with manic onset. Using a twelve-year chart review methodology, we examined the association of first-episode polarity on timing of diagnosis and related outcomes of bipolar I disorder.

## Methods

This study included patients admitted to the Department of Psychiatry, Seoul National University Hospital (Seoul, Korea) over the course of twelve years. Two board-certified psychiatrists reviewed the medical records of patients having a final diagnosis of bipolar I disorder, which was made at discharge from their admission, according to DSM-III-R or DSM-IV criteria (n=280). Patients with a history of organic brain diseases or neurological diseases, or insufficient clinical information were excluded (n=6). Subjects were divided into two groups according to the polarity of the first episode: those with depressive polarity (FE-D) and those with manic (FE-M).^15^ The first episode was defined by the episode when the major depressive episode or manic episode became evident according to the DSM-III-R or DSM-IV criteria. Cases in which the first episode was mixed or involved rapid cycling were excluded (n=16). The final sample included 258 patients (118 men and 140 women). The mean age of the subjects was 33.6 (±12.9) years, and the mean duration of illness was 7.8 (±8.2) years. This study protocol was approved by the Institutional Review Board of Seoul National University Bundang Hospital.

Data on demographic characteristics, clinical variables, and family histories of mood disorders were collected and analyzed for both groups. Clinical variables included the age of the first episode, the length of time between the first episode and confirmed diagnosis of bipolar disorder, the diagnoses given in previous psychiatric admissions, the number of psychiatric hospitalizations prior to the index admission, the histories of suicide attempts, and the polarity of the index episode. When patients reported previous hospitalizations at other hospitals, diagnoses were ascertained by reviewing the referral note or the medical certificate issued by the hospitals at which the diagnoses were made. Self-injuries that were not actual suicide attempts were excluded from the analysis. The time lapse between the first episode and the first suicide attempt was assessed.

The two groups were compared for differences in demographic and clinical characteristics using the chi-square test or Fisher's exact test for categorical variables and the t-test for continuous variables. Because the duration data were not symmetric, we additionally applied a Mann-Whitney U test for comparison of time lag before the bipolar diagnosis between the two groups. All tests were two-tailed with a significance level of 0.05. All statistical procedures were performed using Statistical Package for Social Science (SPSS) 15.0 for Windows (SPSS Inc., Chicago, IL).

## Results

Among the 258 subjects, the FE-M subjects outnumbered (n=179, 69.4%) the FE-D subjects (n=79, 30.6%). No significant differences were observed with regard to demographic characteristics (e.g., age, sex, education, occupation, and marital status) between the two groups (Table 1).

### Time lapse before diagnosis of bipolar disorder

Table 2 shows the clinical characteristics of patients with bipolar I disorder according to the first-episode polarity. No significant differences were observed between the two groups with regard to age at the first episode, age at initial diagnosis of bipolar disorder, and family history of mood disorders. On average, it took 3.5 (±5.8) years to confirm a diagnosis of bipolar I disorder following the first major mood episode. Approximately one-fourth of the total sample waited 5 or more years before receiving the diagnosis of bipolar I disorder, and 12.4% waited 10 or more years from the first major mood episode. FE-D subjects experienced significantly longer intervals between the first episode and initial diagnosis of bipolar I disorder than FE-M subjects [5.6 (±6.1) vs. 2.5 (±5.5) years; t=-4.0, p<0.001]. When a nonparametric analysis was applied, the differences in intervals between the two groups also were significant (Mann-Whitney U=3670.0, p<0.001). The percentage of subjects whose diagnoses were made after 5 or more years was also significantly higher in FE-D group than in FE-M group (41.8% and 18.4%, respectively; χ^2^=42.48, p<0.001).

### Suicide and psychiatric hospitalization

The frequency of suicide attempts was significantly higher in the FE-D group than in the FE-M group (χ^2^=13.82, p=0.001), and the frequency of attempting suicide less than 3 years after the first episode was also significantly higher in FE-D subjects than in FE-M subjects (χ^2^=16.41, p<0.001). Significantly more psychiatric hospitalizations were identified in the FE-M group compared to the FED group (t=2.947, p=0.004). Across all 258 subjects, 177 (68.6%) had experienced at least one psychiatric admission prior to the index admission (Table 2).

### Previous diagnoses according to the first-episode polarity

As shown in Table 3, psychiatric diagnoses prior to the index admission were significantly different according to first-episode polarity (χ^2^=43.96, p<0.001). Most FE-D subjects (n=39, 78%) were diagnosed with psychiatric disorders other than bipolar disorder, most commonly schizophrenia (30%) and major depressive disorder (28%). However, most FE-M subjects (n=77, 61%) were initially diagnosed with bipolar disorder, with schizophrenia as the next common diagnosis.

## Discussion

A substantial delay from the first major mood episode to the initial diagnosis of bipolar I disorder was observed in our subjects. This finding is in line with previous studies,^1^,^2^,^5^ though it took a relatively short period of time to diagnosis of bipolar disorder in our study,^1^,^4^-^6^ compared to results previously reported from Western countries. This difference may be explained by the fact that our study included only inpatients, with presumably more severe bipolar I disorder, and by our adoption of the first major mood episode instead of 'onset of illness', excluding brief or minor mood episodes. Nonetheless, one-fourth of our subjects required 5 or more years before receiving a correct diagnosis of bipolar disorder after experiencing a major mood episode.

The results of this retrospective study also suggest that the polarity of the first episode may be an important contributor to the time lapse before the initial diagnosis of bipolar I disorder. The FE-D group needed twice as much time than the FE-M group to be correctly diagnosed. Previous studies have suggested that depressive episodes occur more frequently when a bipolar disorder starts with a depressive episode,^14^,^15^ and vice versa.^19^ Rosa et al.^13^ reported that the predominance of a depressive polarity can be an important factor in the postponed diagnosis of bipolar disorder. Our results suggest that depressive polarity of the first episode may also be related to the postponed diagnosis of bipolar disorder. Several explanations may be plausible for the postponed diagnosis of bipolar disorder in the FE-D subjects. When bipolar disorders start with a depressive episode followed by recurrent major depressive episodes with inter-episodic subsyndromal depressive symptoms, it is highly likely that the patient will be incorrectly diagnosed with major depressive disorder, especially when manic or hypomanic episodes are not typical or prominent. This is partly due to unavoidable shortcomings of DSM-IV criteria which mandate the presence of manic or hypomanic episodes for the diagnosis of bipolar disorders. Moreover, it is not always possible for psychiatrists to explore possible manic or hypomanic symptoms for all depressed patients in busy clinical settings.^20^ Some patients with bipolar disorders and their care-givers fail to report their manic or hypomanic symptoms to psychiatrists, as they do not regard them as pathologic symptoms. Sometimes the depressive preponderance prevents patients from remembering past manic or hypomanic episodes.^21^ Further research should address the relative contribution of these possibilities to the diagnostic delay in FE-D subjects. The postponed diagnosis of bipolar disorder may lead to unopposed antidepressant exposure in these patients with hidden bipolarity, raising the risk of cyclic acceleration^9^,^22^ or suicidality.^23^ It would be of clinical benefit if we can differentiate bipolar patients among depressed patients earlier in their illness. Recent reports suggest that there may be subtle differences in history and clinical features between unipolar and bipolar depression, including age of onset, number of recurrences, family history of mood disorders, and nature of depressive episode such as atypical features.^9^,^24^,^25^ Another strategy in identifying truly bipolar individuals earlier in their illness is to apply screening tools such as the Mood Disorder Questionnaire (MDQ)^26^ or the Bipolar Spectrum Diagnostic Scale (BSDS).^27^

Delays in confirmation of a correct diagnosis may raise the risk for bipolar I patients to have other, possibly inaccurate psychiatric diagnoses. 34.5% of all our subjects or 50.3% of subjects who had previous psychiatric admission had psychiatric diagnoses other than bipolar disorder, which was made during their previous admissions. This result is in line with previous studies reporting that 46-69% of patients may have other psychiatric diagnosis prior to an accurate diagnosis of bipolar disorder.^28^,^29^ Even though a previous diagnosis such as major depressive disorder may have been reasonable in light of DSM criteria, the diagnosis can be changed to bipolar disorder with subsequent manic or hypomanic episodes, rendering the previous diagnosis as "misdiagnosis" eventually. Like most other countries, bipolar I patients in Korea were at high risk of "misdiagnosis" early in their illness, even though their psychopathology was severe enough to require hospitalization. This may raise the possibility that bipolar I patients had a higher risk of misdiagnosis when their outpatient diagnoses were also taken into consideration. Bipolar II patients may have an even higher risk of misdiagnosis in their early phase when considering their relatively less severe cross-sectional psychopathology compared to bipolar I patients. These possibilities should be investigated at various clinical settings in Korea in order to fully characterize the diagnostic situation, providing information critical to the development of better clinical solutions for diagnosis and management of bipolar disorders. In this study we observed that the type and rate of prior misdiagnoses were also dependent on the type of polarity of the first episode. The FE-D subjects had a far higher risk of misdiagnosis (78.0%) than the FE-M subjects (39.4%). The FE-D subjects had the risk of misdiagnosis with schizophrenia and unipolar at a similar rate, whereas most FE-M subjects had the chance of misdiagnosis with schizophrenia. We can assume that the bipolar patients were at risk of misdiagnosis with schizophrenia when they had associated psychotic symptoms regardless of their polarity, and they were at risk of misdiagnosis of unipolar depression when they presented non-psychotic depressive symptoms. Ghaemi et al.^4^,^30^ also reported that approximately 40% of patients with bipolar disorder had been previously diagnosed with unipolar depression. Since depressive episodes of bipolar disorders can be presented as various subtypes, further studies are warranted to investigate the influence of subtypes to the timing and type of diagnosis.

It is well documented that the predominance of depressive symptoms is strongly related with higher incidence of suicide attempts,^13^,^19^ and Baldessarini et al.^31^ demonstrated that suicide attempts are more frequent during depressive episodes than during manic or hypomanic episodes in bipolar disorders. Our findings showed that the FE-D subjects had a higher rate of suicide attempts compared to the FE-M subjects, which suggests that the depressive polarity of the first episode itself may underlie the tendency towards suicidal behavior in subjects with bipolar I disorder. The FE-D may be related with subsequent frequent depressive episodes and misdiagnosis in the early phase of bipolar disorder which can lead inappropriate initial management. This speculation is also supported by the finding that most of the suicide attempts made by our subjects had occurred within 3 years after the first major mood episode, during the period that most of the subjects were not properly diagnosed and managed for bipolar disorders. Goodwin et al.^32^ reported that lithium may have anti-suicidal effects, and thus can reduce deaths caused by suicide. It seems that vigorous and systematic diagnostic assessment and proper management including lithium for bipolar disorders in their early phase can reduce the risk for suicide attempts, especially for those with initial depressive symptoms.

This study has the following limitations. First, the sample was biased to inpatients with bipolar I disorder at a tertiary level university hospital, suggesting that most patients had experienced mood episodes with psychotic features or severe symptoms that were refractory to conventional treatment. Further studies with representative subjects from the community or clinics, especially non-psychotic outpatients with bipolar I or II disorders, will provide additional useful information from the field. Second, as is often the case with the retrospective chart review methodology, the clinical significance of this study may be limited due to the lack of the interviews with patients or their care-givers. Third, the first episode was defined as the episode that met criteria for major mood episodes, excluding possible minor or brief mood episodes before the major episode. However, exploration of such minor or brief mood episodes is usually very difficult and unrealistic by either retrospective or prospective design. Despite these limitations, the main findings of the current study are in line with the results of previous investigations, and suggest that the depressive polarity of the first episode contributes to extensive delays in accurate diagnosis, a higher rate of other psychiatric diagnoses, and a higher rate of suicide attempts. A systematic diagnostic approach is justified to identify bipolar patients among subjects with depressive episodes, especially in their early phase.

## Figures and Tables

**TABLE 1:**
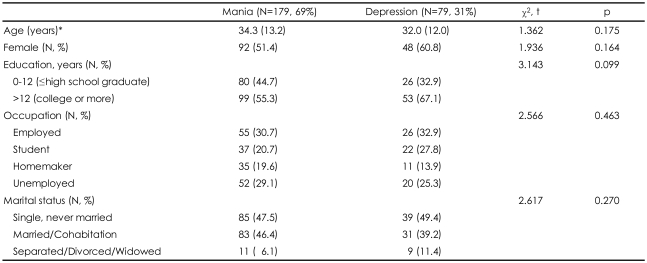
Demographic characteristics of the subjects according to the first-episode polarity

^*^Mean±standard deviation

**TABLE 2:**
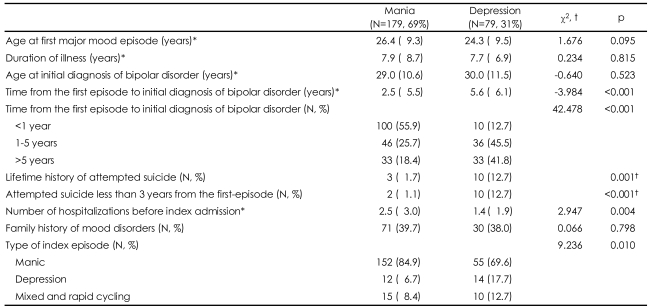
Clinical characteristics of the subjects according to the first-episode polarity

^*^Mean±standard deviation, ^†^Fisher's exact test was done

**TABLE 3:**
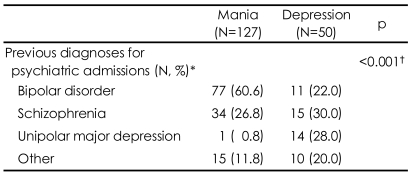
Previous diagnoses of patients with bipolar disorder according to first-episode polarity

^*^Patients who were admitted at least once prior to analysis of the index admission, ^†^Fisher's exact test was done
